# Epidemiology and Genetic Evolutionary Analysis of Influenza Virus Among Children in Hainan Island, China, 2021–2023

**DOI:** 10.3390/pathogens14020142

**Published:** 2025-02-03

**Authors:** Meng Chang, Shengjie Shi, Yan Jin, Gaoyu Wang, Ruoyan Peng, Jing An, Yi Huang, Xiaoyuan Hu, Chuanning Tang, Yi Niu, Xiuying Tian, Wanxin Deng, Cheng Tang, Xiuji Cui, Jasper Fuk-Woo Chan, Yibo Jia, Feifei Yin

**Affiliations:** 1 Department of Clinical Laboratory, Center for Laboratory Medicine, Hainan Women and Children’s Medical Center, Hainan Medical University, Haikou 570206, China; hellochangmeng@163.com (M.C.); 15874843023@163.com (S.S.); a13876068647@hotmail.com (J.A.); dengwanxin2021@163.com (W.D.); 2 Hainan Medical University—The University of Hong Kong Joint Laboratory of Tropical Infectious Diseases, Key Laboratory of Tropical Translational Medicine of Ministry of Education, School of Basic Medicine and Life Sciences, Hainan Medical University, Haikou 571199, China; wgy1205@hainmc.edu.cn (G.W.); pry0302@163.com (R.P.); huangyi930925@163.com (Y.H.); 15300213416@163.com (X.H.); chuanningtang@hotmail.com (C.T.); niuyihnyxy@163.com (Y.N.); tsc1104@163.com (C.T.); cuixj26@163.com (X.C.); 3 Hainan Provincial Center for Disease Control and Prevention, Haikou 571129, China; jinyanfangyi@sina.com; 4 Key Laboratory of Emergency and Trauma of Ministry of Education, Engineering Research Center for Hainan Biological Sample Resources of Major Diseases, The Hainan Branch of National Clinical Research Center for Cancer, The First Affiliated Hospital, Hainan Medical University, Haikou 570102, China; tianxiuying126@163.com; 5 Department of Pathogen Biology, Hainan Medical University, Haikou 571199, China; 6 State Key Laboratory of Emerging Infectious Diseases, Department of Microbiology, and Carol Yu Centre for Infection, Li Ka Shing Faculty of Medicine, The University of Hong Kong, Pokfulam, Hong Kong 999077, China; jfwchan@hku.hk; 7 Department of Infectious Diseases and Microbiology, The University of Hong Kong–Shenzhen Hospital, Shenzhen 518053, China; 8 Medical Administration Division, Hainan General Hospital, Hainan Affiliated Hospital of Hainan Medical University, Haikou 570311, China

**Keywords:** influenza virus, COVID-19, epidemiology, children, Hainan Island

## Abstract

Background: During the COVID-19 pandemic, we continuously monitored the epidemiology of influenza virus among pediatric patients from January 2021 to December 2023 in Hainan Island, China. Methods: In this study, we collected 54,974 nasopharyngeal swab samples for influenza A Virus (IAV) testing and 53,151 samples for influenza B Virus (IBV) testing from pediatric outpatients. Additionally, we also collected 19,687 nasopharyngeal swab samples from pediatric inpatients for IAV and IBV testing. Outpatient samples were screened for influenza viruses (IVs) infection by the colloidal gold method. Targeted Next-Generation Sequencing (tNGS) was used to detect influenza virus infections in inpatients. Influenza virus types were identified by analyzing the HA/NA partial regions. Results: The findings revealed a significant decrease in the infection rate of IBV over the specified period, while the infection rate of IAV exhibited a rising trend. Additionally, B/Victoria lineage was the dominant epidemic strain in 2021, while the epidemic strains in 2022 and 2023 underwent a dynamic transformation from A/H3N2 to A/H1N1. Phylogenetic analysis revealed close relationships among the circulating strains. Nonetheless, because the sample size is limited, additional research is required. Conclusions: Our findings suggest that the predominant types of influenza viruses in the pediatric population are undergoing dynamic changes, influenced by the implementation and relaxation of non-pharmaceutical intervention measures. These findings highlight the need for adaptive influenza vaccination and containment strategies, particularly in tropical regions like Hainan, where climate and public health policies significantly impact viral transmission patterns. The insights gained from this study could inform more effective public health strategies in similar regions to mitigate the impact of influenza outbreaks in the future.

## 1. Introduction

The influenza virus (IV), belonging to the Orthomyxoviridae family, is an RNA virus that can induce acute respiratory infections, exhibiting both seasonal and epidemic attributes [1]. To date, circulating influenza viruses can be categorized into four overarching classifications: influenza A virus (IAV), influenza B virus (IBV), influenza C virus (ICV), and influenza D virus (IDV) [2]. Among these, IAV and IBV are mainly responsible for impact on public health, with IAV provoking more severe clinical diseases and seasonal epidemics in humans [3,4]. Influenza epidemics occur worldwide annually, and are estimated to result in more than 3 to 5 million infections and approximately 291,000 to 646,000 deaths each year [5,6]. It is noteworthy that infections among children make a substantial contribution to the global burden of disease and mortality during the influenza season. Each year, millions of children worldwide experience influenza infections, and it is estimated that between 9243 and 105,690 respiratory deaths annually are attributed to seasonal influenza among children under 5 years of age [5,7].

At the end of 2019, the coronavirus disease 2019 (COVID-19) broke out suddenly and spread rapidly across the world. To prevent and control the epidemic of COVID-19, many countries have adopted strong non-pharmaceutical intervention (NPI) measures, which have changed the lifestyles of the majority of people. Since then, the Chinese government has always adhered to a dynamic zero-COVID policy and implemented a range of measures aimed at curtailing the further dissemination of the virus, including lockdowns, the closures of schools, and social isolation. During the COVID-19 pandemic, although the influenza diagnosis rate varies in space and time in different countries, the significant decline compared with the pre-epidemic level is global [8]. However, the extent of this decline varied among regions [9,10]. Meanwhile, China adhered to a dynamic zero-COVID policy until 7 December 2022, after which a notable resurgence of influenza cases was observed in several areas [11]. Hainan Island—China’s only tropical island province—presents a unique environment for studying influenza activity due to its year-round warm and humid climate. Hence, we examined the influenza epidemiology and genetic evolution among children in Hainan during the transition from dynamic zero-COVID to reopening, aiming to provide critical insights into future preventive measures.

Typically, climate factors can affect the spread of viral diseases. While temperate climate regions typically experience influenza epidemics during the winter months, tropical climate regions have infections occurring all year round, leading to more unpredictable outbreaks [12]. Hainan is the only tropical island province in China, located at the southernmost point of China and isolated from the Chinese mainland, experiences a relatively high annual average temperature range of 22.5 °C to 25.6 °C, and maintains a consistently humid climate throughout the year. Due to its unique geographical location and climate characteristics, Hainan Island may have even greater challenges regarding viral transmission. Consequently, we carried out a study on the epidemiology and genetic evolution of influenza among children in Hainan Island during the shift phase from dynamic zero-COVID to reopening. Our goal was to gather valuable insights, bolster our readiness for potential influenza outbreaks, and devise more effective preventive measures.

## 2. Methods

From January 2021 to December 2023, we collected 54,974 nasopharyngeal swab samples from pediatric outpatients at the Hainan Women and Children’s Medical Center for IAV testing and 53,151 samples for IBV testing. And 19,687 nasopharyngeal swab samples from pediatric inpatients were collected for IAV and IBV testing. The Hainan Women and Children’s Medical Center is a provincial-level non-profit medical institution, undertaking most of the disease burden for women and children in Hainan province, as well as the responsibility of health care and education for women and children. This study was approved by the Ethics Committee of Hainan Medical University. Nasopharyngeal swabs were collected during a patient at presentation or admission and stored at −80 °C. Outpatient samples were screened for IV infection by the colloidal gold method. Targeted Next-Generation Sequencing (tNGS) supported by KingMed Diagnostics was used to detect IV infection from inpatients [13,14].

During this study period, we sequenced amplification sequencing on the HA and NA regions of 21 influenza isolates obtained at different stages, which were identified as A(H1N1)pdm09, A(H3N2), and B(Victoria). These 21 isolates were selected to capture the genetic diversity of circulating influenza viruses across various peak incidence periods from 2021 to 2023, ensuring a comprehensive representation of the major subtypes in the pediatric population on Hainan Island [15]. This sample size is comparable to similar surveillance studies, where the focus is on revealing key evolutionary patterns rather than providing an exhaustive inventory of all circulating strains.

To ensure a comprehensive phylogenetic analysis, IV isolates were chosen from various influenza prevalent periods, using primer information from WHO [16]. These isolates were amplified by PCR. The sequencing primers and PCR conditions for the HA and NA regions are detailed in Table S1.The 21 IV-positive samples from inpatients were strategically selected based on their collection times from 2021 to 2023. While 21 isolates may not capture the entire spectrum of genetic diversity, this approach provides critical insights into the evolutionary trends of the virus on Hainan Island. The PCR products were sequenced by Sanger sequencing at the Tsingke Biotechnology Co., Ltd. (Beijing, China). The sequencing results were edited in SeqMan software (version 7.1.0) and identified using the BLAST from the National Center for Biotechnology Information (NCBI). Phylogenetic analyses were conducted on IAV and IBV using specific gene segments. For IAV, the analysis included A/H1N1 based on a 1131 bp segment of the HA gene and a 906 bp segment of the NA gene, as well as A/H3N2 based on a 754 bp segment of the HA gene and a 926 bp segment of the NA gene. For IBV, the analysis included B/Victoria based on a 529 bp segment of the HA gene and a 393 bp segment of the NA gene. Reference strains were selected based on their relevance to the circulating strains in Hainan Island from 2021 to 2023. Additionally, we selected sequences of influenza strains circulating prior to the pandemic from public databases, focusing on strains from various regions in China, including central and southern China, and specifically Hainan. Furthermore, we included strains from Russia, Tajikistan, Thailand, Japan, Cameroon, Togo, Egypt, South Sudan, Chile, the United States, and regions such as Hong Kong. These strains were obtained from the GISAID database, ensuring a comprehensive representation of the genetic diversity and evolutionary trends of the IVs [17]. Analysis of the sequences was performed using BioEdit software (version 7.0.5.3). The sequencing data were submitted to the NCBI under accession number, from PP732243 to PP732252, PP732220 to PP732225, PP732456 to PP732464, and PP736112 to PP736121.

Data were analyzed using the Chi-square (χ^2^) test in SPSS software (version 25.0). Statistical significance was determined at a threshold of *p* < 0.05. Phylogenetic trees were constructed using neighbor-joining and bootstrapping of 1000 replications in MEGA X software (version 10.2.2). Origin 2021 software was used to draw the figures.

## 3. Results

### 3.1. Sample Information and Demographics

During the study period, a total of 54,974 pediatric outpatients underwent IAV testing, and the IBV test population numbers of pediatric outpatients were 53,151 (Table 1). All the pediatric patients were admitted to Hainan Maternal and Child Health Hospital. The age range of participants for IAV and IBV included individuals aged 0 to 18 years (Table 2). The demographic details of the cases have been summarized in Table 1 and Table 2. In the IAV testing, the median (interquartile, IQ) age is 4.98 years (2.58–7.71), including 32,162 males (58.50%) and 22,812 females (41.50%). The IBV test population numbers of outpatient children were 53,151, consisting of 31,123 males (58.56%) and 22,028 females (41.44%), with a median (IQ) age of 5.07 years (2.61–7.77). Nasopharyngeal swab specimens were also collected from hospitalized children with respiratory illness. The median age of the 19,687 pediatric inpatients was 2.34 years (1.08–5.06), comprising 12,131 males (61.62%) and 7556 females (38.38%). Furthermore, the number of samples collected for IAV antigen detection in 2021, 2022, and 2023 were 7728, 16,464, and 30,782 cases, accounting for 14.06%, 29.95%, and 55.99% of total outpatient visits, respectively. The number of samples collected for IBV antigen detection were 7812, 16,956, and 28,383 cases from 2021 to 2023, representing 14.70%, 31.90%, and 53.40% of the total outpatient visits, respectively. The number of influenza detection samples collected from hospitalized patients were 4721 cases (23.98%), 5227 cases (26.55%), and 9739 cases (49.62%) in 2021, 2022, and 2023, respectively.

### 3.2. Epidemiology of Influenza Among Children in Hainan from 2021 to 2023

During the period of this study, the overall positive rates of IAV among outpatients and inpatients were 28.54% and 5.02%, respectively. The positive rates of IBV among outpatients and inpatients were 5.41% and 1.64%, respectively. Annual incidence rates of IAV and IBV among outpatients and inpatients are represented in Table 1, showing statistically significant differences (*p* < 0.05). There was a noticeable increase in the positive rate of IAV for 2022 and 2023 compared to 2021 (outpatients: χ^2^ = 3171.13, *p* < 0.001, inpatients: χ^2^ = 320.18, *p* < 0.001). Additionally, the positive rate of IBV among outpatients in 2023 was higher than in 2022, but lower than in 2021 (χ^2^ = 3681.40, *p* < 0.001). For inpatients, there were no statistically significant differences in the positive rate of IBV between 2023 and 2022, and both rates were lower than in 2021(χ^2^ = 182.71, *p* < 0.001). In the intensive care unit (ICU), IAV positive cases accounted for 0.00% (0/3), 0.11% (33/313), and 0.06% (39/648) from 2021 to 2023 among pediatric inpatients. The number of IBV-positive inpatients in the ICU was 5, 1, and 7 cases from 2021 to 2023, accounting for 0.03%, 0.02%, and 0.07% of the total IBV-positive inpatient infections, respectively.

**Figure 1:**
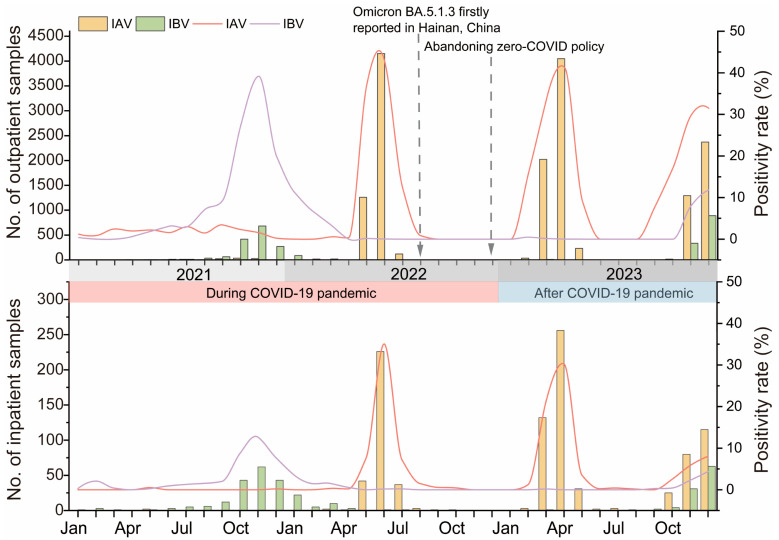
Children on Hainan Island, China, during 2021–2023. The upper panel illustrates the monthly number of pediatric outpatients tested and the influenza-positive cases, while the lower panel presents the same information for pediatric inpatients. Key time points include the dynamic zero-COVID period (1 January 2021 to 7 December 2022) and the first detection of the Omicron BA.5.1.3 variant in Hainan Province on 1 August 2022. The dashed vertical line indicates the end of the dynamic zero-COVID policy. Seasonal trends can be observed by tracking the monthly distribution of total samples tested and the proportion of positive cases. Abbreviations: No., number; IAV, influenza A virus; IBV, influenza B virus; Jan, January; Apr, April; Jul, July; Oct, October.

**Figure 2:**
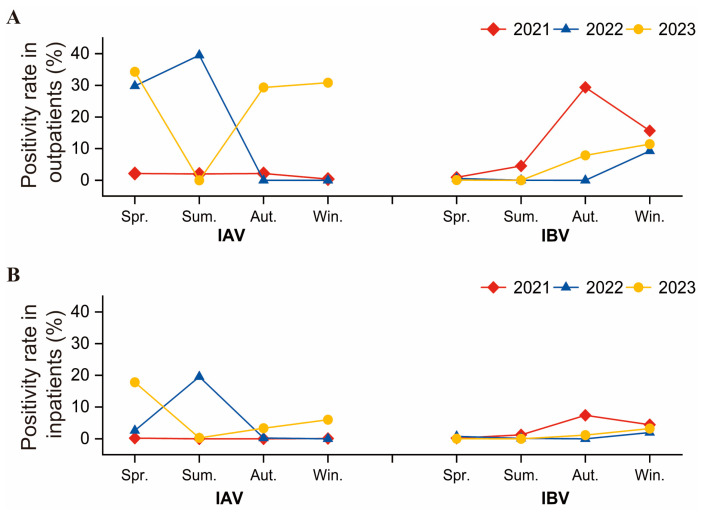
Positive rate of IAV and IBV by season group among children in Hainan Island, China, during 2021–2023. (**A**) IAV and IBV data analysis among pediatric outpatients. (**B**) IAV and IBV data analysis among pediatric inpatients. Note: The red line shows the seasonal positive rate in 2021, the blue line in 2022, and the yellow line in 2023. These color codes allow for an easy comparison of seasonal patterns across three consecutive years. Abbreviations: IAV, influenza A virus; IBV, influenza B virus; Spr., spring; Sum., summer; Aut., autumn; Win., winter.

Figure 1 illustrates the monthly distribution of IAV and IBV infections among both outpatients and inpatients from January 2021 to December 2023. While the overall trends were similar in both groups, the rate of infections was consistently higher among outpatients. Specifically, influenza activity remained subdued until October 2021, when IBV-positive rates began to rise, peaking in November, followed by a decline in influenza activity. The IBV infection rate was 39.16% in outpatients and 12.63% in inpatients in November. In 2021, the wave of influenza activity was primarily concentrated in autumn, dominated by IBV infections, and only a small number of IAV cases were detected. The positive rate of IAV increased obviously in May 2022, reached its peak in June, and decreased drastically in July, with an IAV positivity rate of 42.78% among outpatients and 35.04% among inpatients in June. Notably, there was almost no influenza activity in the following six months. In 2022, different from the previous year, influenza activity was obvious in summer with the predomination of IAV, while no influenza activity was observed in autumn. Influenza activity did not appear until a high level of IAV was detected in March 2023, reaching its peak in April and declining rapidly in May. In April 2023, there was an IAV infection rate of 41.26% in outpatients and 30.26% in inpatients. During this wave, the influenza activity escalated and then declined in spring, which was two months earlier than the previous year. From June to September 2023, influenza activity remained minimal until a slight increase in IAV and IBV activity in October 2023. Subsequently, high levels of IAV and IBV were detected from November to December 2023, indicating a prolonged influenza wave. Moreover, in December 2023, 31.45% of outpatients and 8.03% of inpatients tested positive for IAV, while 11.82% of outpatients and 4.40% of inpatients tested positive for IBV. Regarding the second wave in 2023, the influenza activity commenced in autumn and winter, starting a month later than in 2021. Throughout 2023, two noticeable influenza outbreaks occurred, one in spring predominated by IAV and another in autumn and winter, characterized by the simultaneous transmission of IAV and IBV, with minimal activity in summer. We observed notable seasonal variations in influenza activity, with IAV peaks in summer 2022 and spring 2023, likely influenced by public health interventions and Hainan’s tropical climate. These findings emphasize the importance of adaptive public health strategies, including flexible vaccination campaigns and continuous surveillance, especially in tropical regions where year-round transmission is possible. Over the three-year study period, the prevalence of influenza seemed to lack a consistent seasonal pattern. As depicted in Figure 2, the outpatient IBV positivity rate reached 29.39% in autumn 2021, while the outpatient IAV positivity rate reached 39.55% in summer 2022 and exceeded 25% in spring, autumn, and winter 2023. To analyze the variation in the activity intensity of IAV and IBV during the study period, we compared the positivity rate changes in the same season over the three years. In 2023, the activity intensity of IAV in spring, autumn, and winter was significantly higher than in the corresponding seasons of 2021 and 2022 (spring: outpatients: χ^2^ = 388.99, *p* < 0.001, inpatients: χ^2^ = 388.50, *p* < 0.001; autumn: outpatients: χ^2^ = 1192.68, *p* = 0.00, inpatients: χ^2^ = 74.25, *p* < 0.001; winter: outpatients: χ^2^ = 1126.17, *p* < 0.001, inpatients: χ^2^ = 146.41, *p* = 0.00), while in summer, it was notably lower than in 2021 and 2022 (outpatients: χ^2^ = 760.61, *p* < 0.001, inpatients: χ^2^ = 671.77, *p* < 0.001). Regarding influenza B, the activity intensity in autumn 2023 was markedly higher than in 2022 but lower than in 2021 autumn (outpatients: χ^2^ = 712.57, *p* = 0.00, inpatients: χ^2^ = 175.78, *p* < 0.001).

**Figure 3:**
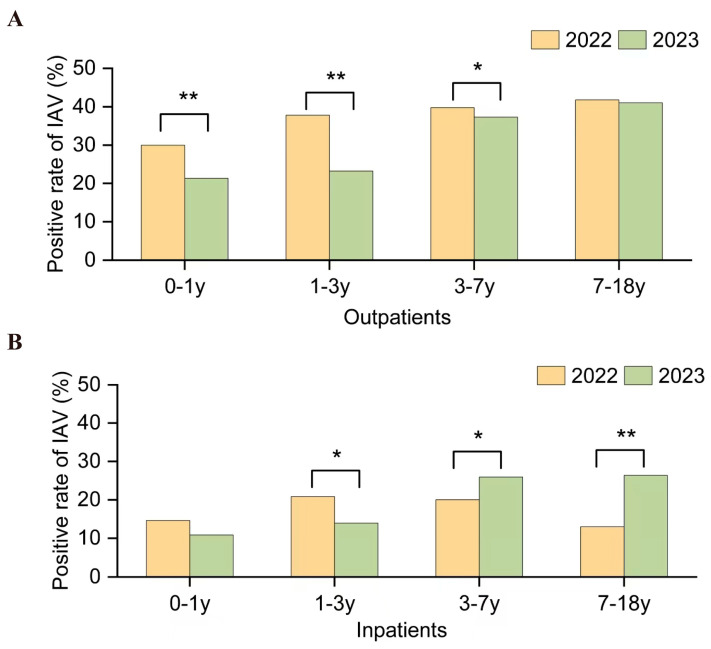
Comparison of IAV-positive rate by age group among children on Hainan Island between May to July 2022 and March to May 2023. (**A**) Data analysis among pediatric outpatients. (**B**) Data analysis among pediatric inpatients. Note: Age groups are defined as 0–1 year (infants), 1–3 years (early childhood), 3–7 years (preschool), and 7–18 years (school-age). Vertical bars colored in yellow represent data from May to July 2022, while green bars represent data from March to May 2023. The asterisks (*, **) indicate significant differences (*p* < 0.05, *p* < 0.001, respectively) in the positive rate between the two time periods. The color scheme highlights any shifts in positivity across these age groups.

There was no significant difference in the detection rates of IAV and IBV between genders each year (Table 2, *p* > 0.05). To investigate susceptibility differences among different age groups, the ages were categorized into four groups: 0–1 year (infants), 1–3 years (early childhood), 3–7 years (preschool children), and 7–18 years (school-age children). In 2021, no differences were observed in the detection rates of IAV among hospitalized children in different age groups. However, statistical variances were noted among different age groups in the detection rates of IAV and IBV in other settings (Table 2, *p* < 0.05). In 2021, there were no differences in the detection rates of IAV among outpatient infants, preschool children, and school-age children, while the detection rate of IBV was highest among school-age children in both outpatients and pediatric inpatients, followed by preschool children. In 2022 and 2023, the highest detection rates of IAV and IBV were observed in school-age children among outpatients, followed by preschool children. Before and after the transition from the dynamic zero-COVID policy, two waves of IAV activities occurred, one from May to July 2022 and another from March to May 2023. Comparing the IAV positivity rates during these periods revealed higher rates in infants, early childhood, and preschool children among outpatients in 2022 compared to 2023 (0–1: χ^2^ = 25.34, *p* < 0.001; 1–3: χ^2^ = 155.86, *p* < 0.001; χ^2^ = 8.76, *p* = 0.003); however, no significant differences were observed in school children (Figure 3). Conversely, the IAV-positive rates in preschool children and school children among inpatients were lower in 2022 than in 2023 (3–7: χ^2^ = 6.32, *p* = 0.01, 7–18: χ^2^ = 15.60, *p* < 0.001).

### 3.3. Phylogenetic Characterization of IAV and IBV in Hainan During 2021–2023

**Figure 4:**
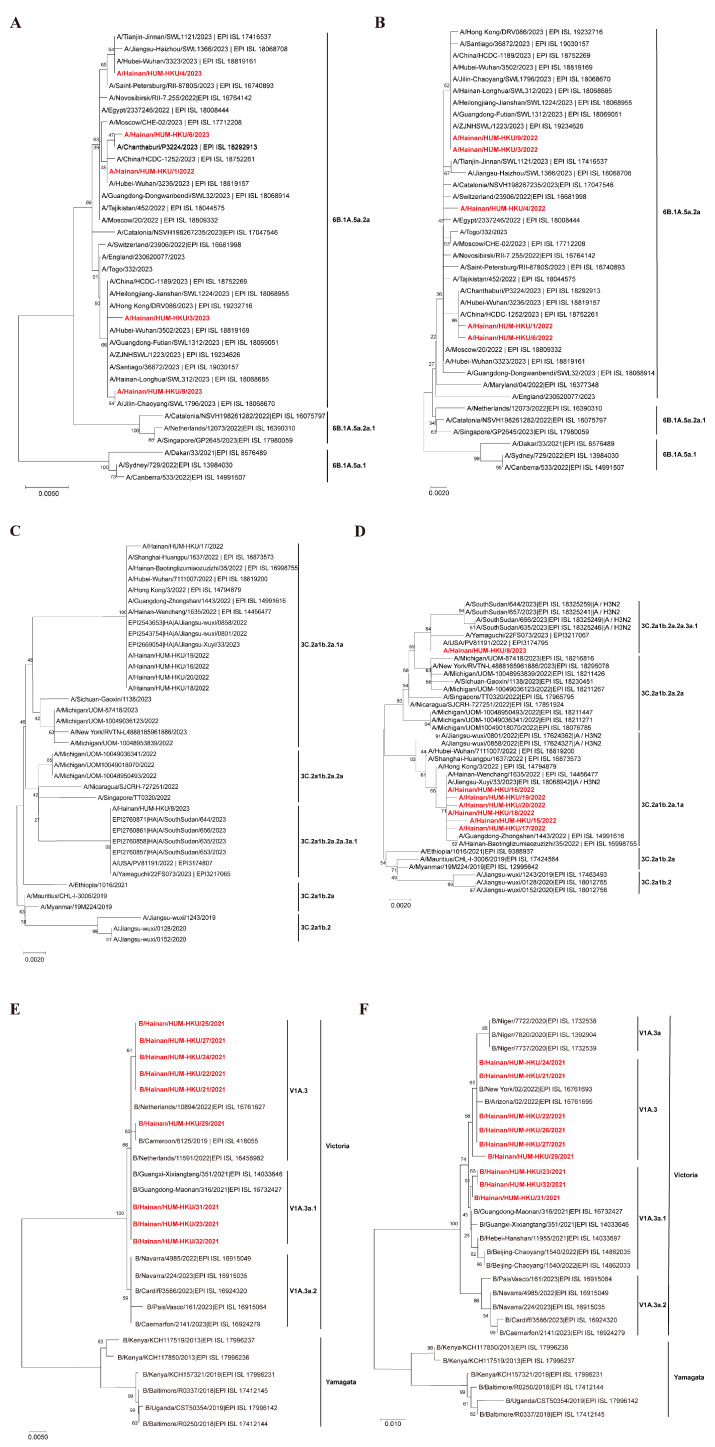
Phylogenic tree of IAV and IBV among children on Hainan Island, China, from 2021 to 2023. (**A**) Phylogenic analysis of A/H1N1 based on the HA gene. (**B**) Phylogenic analysis of A/H1N1 based on the NA gene. (**C**) Phylogenic analysis of A/H3N2 based on the HA gene. (**D**) Phylogenic analysis of A/H3N2 based on the HA gene. (**E**) Phylogenic analysis of B/Victoria based on the HA gene. (**F**) Phylogenic analysis of B/Victoria based on the NA gene. Note: Sequences from this study are shown in red and reference strains are shown in black. The scale bar corresponds to genetic distance, expressed as the number of base substitutions per site. Highlighting our study isolates in red helps readers quickly differentiate them from global reference strains.

During our study period, we conducted PCR amplification and sequencing on the HA and NA regions from 21 influenza isolates obtained at various stages, yielding three types: A(H1N1)pdm09, A(H3N2), and B(Victoria). These isolates were strategically selected from multiple peak incidence periods between 2021 and 2023 to capture major subtypes and reflect the genetic diversity circulating in Hainan’s pediatric population. In sequencing the IAV samples, approximately 1300 bp of the HA fragment and 1100 bp of the NA fragment of H1N1 were obtained, covering about 16.33% to 17.66% of the H1N1 genome. For H3N2, approximately 900 bp of the HA fragment and 1100 bp of the NA fragment were obtained, covering about 13.79% to 14.81% of the H3N2 genome. During the sequencing of the IBV samples, approximately 800 bp of the HA fragment and 400 bp of the NA fragment were obtained, covering approximately 8.28% to 8.39% of the IBV genome. Using reference sequences from databases such as NCBI and GISAID, we constructed six phylogenetic trees based on both HA and NA (Figure 4). Figure 4A,B showed the phylogenetic trees of the HA and NA derived from H1N1, with the isolated strains belonging to 6B.1A.5a.2a, one from 2022 and the others from 2023, showing consistent results. Furthermore, the phylogenetic analysis of the HA and NA genes revealed that all five H3N2 isolates in 2022 clustered within the 3C.2a1b.2a.1a clade, while one H3N2 isolate from 2023 fell within the 3C.2a1b.2a.2a.3a.1 clade (Figure 4C,D). Notably, the genetic evolution delineated a pattern: the 2022 outbreak was predominantly H3N2, with sequence similarities ranging from 98.10% to 100% based on the HA segment, while the 2023 outbreak was majorly H1N1–09, with sequence similarities for the HA segment ranging from 99.30% to 99.8%. The NA segment analysis echoed these findings, showing sequence similarities between 99.40% and 100%. The phylogenetic analysis based on the HA segment in Figure 4E revealed that all circulating IBV strains in Hainan during 2021 clustered within a single branch corresponding to the Victoria lineage, which was further subdivided into two subtypes: V1A.3a.1 and V1A.3. Further sequence comparison indicated a high similarity among these strains, with sequence identities ranging from 99.70% to 100% for the HA segment. The analysis from the NA segment corroborated the HA findings, presenting sequence similarities between 98.70% and 100% (Figure 4F). Our data indicate that Hainan experienced three outbreaks of influenza between 2021 and 2023. In 2021, the epidemic was predominantly caused by B(Victoria). In contrast, the epidemics in 2022 and 2023 were primarily driven by IAV, with A (H3N2) in 2022 and A(H1N1)pdm09 in 2023. Of particular interest is the alternating prevalence of H3N2 and H1N1 genotypes during the IAV epidemics in 2022 and 2023. As one genotype dominated, the incidence of the other significantly reduced but did not vanish entirely, suggesting a dynamic interplay between these genotypes in the region.

## 4. Discussion

This study investigates the epidemiological characteristics and genetic diversity of influenza viruses circulating among children on Hainan Island, both during and following the COVID-19 epidemic. Research consistently demonstrates that children exhibit the highest susceptibility to influenza infection and are at an elevated risk of hospitalization due to their immature immune system [18]. The unique climatic and geographical attributes of Hainan Island, combined with its status as a major tourist destination and a free trade port, significantly influence the transmission dynamics of influenza viruses.

In our findings, Hainan witnessed a significant transition in influenza virus dominance on Hainan Island, shifting from IBV in the winter of 2021 to IAV during the summer months of 2022. In 2023, two waves of influenza occurred, initially dominated by IAV, and subsequently by a concurrent epidemic of IAV and IBV. Remarkably, the volume of influenza detections in 2023 exceeded those recorded in the preceding two years, suggesting a resurgence to pre-pandemic influenza activity levels. This finding highlights how the lifting of non-pharmaceutical interventions (NPIs) significantly influenced the transmission dynamics of influenza. Influenza typically exhibits seasonal epidemics globally, particularly in temperate regions during the winter; however, in tropical settings such as Hainan, activity can persist all year round [19]. In China, variations in the seasonality and evolutionary dynamics across different regions are common. Prior to the COVID-19 pandemic, northern provinces experienced winter influenza A epidemics peaking in January to February, while southern provinces occurred peak viral activity in spring (April to June).

The impact of COVID-19 outbreaks and associated NPIs on influenza activity globally declined in 2020, including China, Asia, the US, and Europe, and gradually rebounded in 2021 [20,21]. Interestingly, our study observed a shift from B-dominant influenza in 2021 to A-dominant or co-endemic waves in 2022 and 2023 following the abandonment of the dynamic zero-COVID strategy. The peak of IAV activity in the summer of 2022 can be attributed to the partial relaxation of NPIs, such as localized lockdowns and reduced social distancing measures [22]. The re-opening of schools and resumption of social activities among children likely contributed to increased influenza transmission due to high levels of social interaction [23]. In contrast, the peak observed in 2023, post the zero-COVID policy, was influenced by the complete lifting of COVID-19 restrictions, leading to a significant increase in social mixing and travel. Furthermore, the lack of prior exposure to influenza viruses during stringent COVID-19 control measures might have reduced population immunity, contributing to a higher susceptibility to influenza infections [24]. Similar rebounds in influenza activity following the relaxation of NPIs have been documented in other regions globally, underscoring the pivotal role of NPIs in shaping population-level immunity. Although influenza activity starting to rebound in 2021, our research revealed a period from August 2022 to January 2023 when influenza activity stalled for over six months without the previous autumn and winter influenza seasons. Our research indicated that school-age children among outpatients were more susceptible to infection, as supported by the majority of other studies focusing on children [25,26].

Before the COVID-19 pandemic, global influenza surveillance showed that both influenza A(H1N1)pdm09, A(H3N2), and B lineages were co-circulating globally, with A(H3N2) often being the predominant cause of seasonal epidemics globally [8,27]. Our study on influenza patterns in Hainan from 2021 to 2023 reflects this shift, with B/Victoria lineage in 2021 and influenza A predominating in subsequent years. The oscillating dynamics between H3N2 and H1N1 in 2022 and 2023 underscore the complexity of influenza evolution, shaped by factors such as antigenic drift, temporary cross-immunity, and viral competition [28]. This study has several limitations. First, only 21 IV-positive samples were sequenced to assess genetic variation, a sample size that—while consistent with similar surveillance studies—may not fully capture the breadth of circulating genotypes. Our strategic selection aimed to include multiple time points and influenza peaks, but a larger dataset would permit more definitive conclusions regarding genetic diversity and evolutionary trends. Second, focusing primarily on hospitalized pediatric cases could underrepresent milder or asymptomatic infections within the wider community, thus potentially introducing selection bias. Future research should prioritize longitudinal studies to monitor the evolution and transmission of influenza viruses over time. Expanding genetic analyses with larger and more diverse sample sets, including community-based and outpatient samples, would provide deeper insights into the genetic diversity and dynamics of influenza in Hainan and similar regions. In addition, integrating genetic surveillance findings with vaccination programs could further optimize vaccine strain selection and enhance public health outcomes. These efforts would help refine our understanding of influenza virus behavior and guide more effective public health strategies.

## 5. Conclusions

This study highlights the profound impact that public health policies have on the epidemiological behavior of influenza. The findings presented are integral to deepening our understanding of influenza epidemiology and its complex interactions with other significant infectious diseases, particularly in the context of major global health events like the COVID-19 pandemic. As we move beyond the immediate impacts of COVID-19, it remains imperative to maintain vigilance and preparedness against the possibility of the simultaneous circulation of multiple respiratory viruses. Continuous research is essential to accurately characterize the evolving epidemiological trends and variations of influenza viruses, thereby informing effective public health strategies and interventions.

## Figures and Tables

**Table 1:** Detection of IAV and IBV from children in Hainan, 2021 to 2023.

Year	Outpatients of IAV (n = 54,974)		*p*	Inpatients of IAV (n = 19,687)		*p*	Outpatients of IBV (n = 53,151)		*p*	Inpatients of IBV (n = 19,687)		*p*
	Positive (%)	Negative		Positive (%)	Negative		Positive (%)	Negative		Positive (%)	Negative	
2021	135_a_ (1.75)	7593		3_a_ (0.06)	4718		1502_a_ (19.23)	6310		180_a_ (3.81)	4541	
2022	5534_b_ (33.61)	10,930	<0.001	337_b_ (6.45)	4890	<0.001	136_b_ (0.80)	16,820	<0.001	42_b_ (0.80)	5185	<0.001
2023	10,021_b_ (32.55)	20,761		648_b_ (6.65)	9091		1236_c_ (4.35)	27,147		101_b_ (1.04)	9638	
Total	15,690 (28.54)	39,284		988 (5.02)	18,699		2874 (5.41)	50,277		323 (1.64)	19,364	

Abbreviation: IAV, influenza A virus; IBV, influenza B virus. % = percentage of positive cases among total samples tested in each group. Pairwise comparisons by year were performed using the Bonferroni method, with subscript letters indicating subsets of influenza outcome categories at the 0.05 significance level. Matching letters within the same column signify no statistically significant difference; different letters indicate a significant difference.

**Table 2:** Annual detection and comparison of outpatient and inpatient cases of IAV and IBV among children based on gender and age in Hainan, 2021–2023.

Parameter	Year		Outpatients of IAV		*p*	Inpatients of IAV		*p*	Outpatients of IBV		*p*	Inpatients of IBV		*p*
			Positive (%)	Negative		Positive (%)	Negative		Positive (%)	Negative		Positive (%)	Negative	
	2021	Male	88 (1.88)	4588	0.262	0 (0.00)	3002	0.091	921 (19.45)	3814	0.533	122 (4.06)	2880	0.234
		Female	47 (1.54)	3005		3 (0.17)	1716		581 (18.88)	2496		58 (3.37)	1661	
Gender	2022	Male	3295 (33.97)	6404	0.242	220 (6.93)	2953	0.075	79 (0.79)	9918	0.836	21 (0.66)	3152	0.154
		Female	2239 (33.10)	4526		117 (5.70)	1937		57 (0.82)	6902		21 (1.02)	2033	
	2023	Male	5886 (33.09)	11,901	0.529	415 (6.97)	5541	0.119	738 (4.50)	15653	0.154	62 (1.04)	5894	0.962
		Female	4135 (31.82)	8860		233 (6.16)	3550		498 (4.15)	11494		39 (1.03)	3744	
	2021	0–1	3_a_ (0.29)	1037	0.001	1_a_ (0.05)	1928	1.000	36_a_ (3.44)	1012	<0.001	33_a_ (1.71)	1896	<0.001
		1–3	40_b_ (2.03)	1935		1_a_ (0.07)	1408		119_b_ (5.95)	1880		40_a_ (2.84)	1369	
		3–7	54_b_ (2.18)	2423		1_a_ (0.09)	1116		409_c_ (16.28)	2103		71_b_ (6.36)	1046	
		7–18	38_b_ (1.70)	2198		0_a_ (0.00)	266		938_d_ (41.63)	1315		36_c_ (13.53)	230	
Age	2022	0–1	327_a_ (23.04)	1092	<0.001	58_a_ (4.10)	1355	0.004	8_a_ (0.55)	1443	<0.001	6_a_ (0.42)	1407	0.005
		1–3	1033_b_ (29.88)	2424		89_b_ (6.82)	1216		16_a_ (0.45)	3536		5_a_ (0.38)	1300	
		3–7	2300_c_ (34.34)	4397		125_b_ (6.91)	1684		38_a_ (0.55)	6867		25_b_ (1.38)	1784	
		7–18	1874_d_ (38.32)	3017		41_a, b_ (5.86)	659		74_b_ (1.47)	4974		6_a, b_ (0.86)	694	
	2023	0–1	476 _a_ (21.53)	1735	<0.001	110_a_ (4.10)	2575	<0.001	30_a_ (1.59)	1851	<0.001	9_a_ (0.34)	2676	<0.001
		1–3	1242_a_ (22.81)	4203		136_a_ (5.69)	2256		115_a_ (2.45)	4575		17_a,b_ (0.71)	2375	
		3–7	4680_b_ (34.54)	8871		276_b_ (8.78)	2867		446_b_ (3.53)	12,184		41_b, c_ (1.30)	3102	
		7–18	3623_c_ (37.84)	5952		126_b_ (8.29)	1393		645_c_ (7.02)	8537		34_c_ (2.24)	1485	

Abbreviation: IAV, influenza A virus; IBV, influenza B virus. % = percentage of positive cases among total samples tested in each group. Pairwise comparisons by year were performed using the Bonferroni method, with subscript letters indicating subsets of influenza outcome categories at the 0.05 significance level. Matching letters within the same column signify no statistically significant difference; different letters indicate a significant difference.

## Data Availability

All sequences analyzed during this study are available from the NCBI database (GenBank accession No. PP732243–PP732252, PP732220–PP732225, PP732456–PP732464, PP736112–PP736121).

## Supplementary Materials

The following supporting information can be downloaded at: https://www.mdpi.com/article/10.3390/pathogens14020142/s1, Table S1: Primers and PCR conditions for the HA and NA regions.

## References

[1] Shie J.J., Fang J.M. (2019). Development of effective anti-influenza drugs: Congeners and conjugates—A review. J. Biomed. Sci..

[2] Uprety T., Sreenivasan C.C., Bhattarai S., Wang D., Kaushik R.S., Li F. (2021). Isolation and development of bovine primary respiratory cells as model to study influenza D virus infection. Virology.

[3] Motta F.C., Born P.S., Resende P.C., Brown D., Siqueira M.M. (2019). An Inexpensive and Accurate Reverse Transcription-PCR-Melting Temperature Analysis Assay for Real-Time Influenza Virus B Lineage Discrimination. J. Clin. Microbiol..

[4] Rcheulishvili N., Papukashvili D., Liu C., Ji Y., He Y., Wang P.G. (2022). Promising strategy for developing mRNA-based universal influenza virus vaccine for human population, poultry, and pigs- focus on the bigger picture. Front. Immunol..

[5] Iuliano A.D., Roguski K.M., Chang H.H., Muscatello D.J., Palekar R., Tempia S., Cohen C., Gran J.M., Schanzer D., Cowling B.J. (2018). Estimates of global seasonal influenza-associated respiratory mortality: A modelling study. Lancet.

[6] Yang W., Lau E.H.Y., Cowling B.J. (2020). Dynamic interactions of influenza viruses in Hong Kong during 1998–2018. PLoS Comput. Biol..

[7] Tasar S., Karadag-Oncel E., Yilmaz-Ciftdogan D., Kara-Aksay A., Ekemen-Keles Y., Elvan-Tuz A., Ustundag G., Sahin A., Kanık M.A., Yilmaz N. (2022). Influenza is more severe than our newest enemy (COVID-19) in hospitalized children: Experience from a tertiary center. J. Med. Virol..

[8] Bonacina F., Boëlle P.Y., Colizza V., Lopez O., Thomas M., Poletto C. (2023). Global patterns and drivers of influenza decline during the COVID-19 pandemic. Int. J. Infect. Dis..

[9] (2023). Wise J Covid-19: WHO declares end of global health emergency. BMJ.

[10] Sominina A., Danilenko D., Komissarov A.B., Pisareva M., Fadeev A., Konovalova N., Eropkin M., Petrova P., Zheltukhina A., Musaeva T. (2023). Assessing the Intense Influenza A(H1N1)pdm09 Epidemic and Vaccine Effectiveness in the Post-COVID Season in the Russian Federation. Viruses.

[11] National Health Commission of the People’s Republic of China Notice. http://www.nhc.gov.cn/xcs/zhengcwj/202212/6630916374874368b9fea6c2253289e1.shtml.

[12] World Health Organization Influenza Seasonal. https://www.who.int/health-topics/influenza-seasonal#tab=tab_1.

[13] Li S., Tong J., Li H., Mao C., Shen W., Lei Y., Hu P. (2023). L. *pneumophila* Infection Diagnosed by tNGS in a Lady with Lymphadenopathy. Infect. Drug Resist..

[14] Xiao M., Banu A., Jia Y., Chang M., Wang G., An J., Huang Y., Hu X., Tang C., Li Z. (2024). Circulation pattern and genetic variation of rhinovirus infection among hospitalized children on Hainan Island, before and after the dynamic zero-COVID policy, from 2021 to 2023. J. Med. Virol..

[15] Dudin G., Aziz I., Alzayed R., Ahmed A., Hussain T., Somily A.M., Alsaadi M.M., Almajhdi F.N. (2023). Genetic Diversity and Evolutionary Kinetics of Influenza A Virus H3N2 Subtypes Circulating in Riyadh, Saudi Arabia. Vaccines.

[16] World Health Organization WHO Information for Molecular Diagnosis of Influenza Virus—Update. https://www.who.int/teams/global-influenza-programme/laboratory-network/quality-assurance/eqa-project/information-for-molecular-diagnosis-of-influenza-virus.

[17] Khare S., Gurry C., Freitas L., Schultz M.B., Bach G., Diallo A., Akite N., Ho J., Lee R.T., Yeo W. (2021). GISAID’s Role in Pandemic Response. China CDC Wkly..

[18] Banzhoff A., Stoddard J.J. (2012). Effective influenza vaccines for children: A critical unmet medical need and a public health priority. Hum. Vaccines Immunother..

[19] Javanian M., Barary M., Ghebrehewet S., Koppolu V., Vasigala V.R., Ebrahimpour S. (2021). A brief review of influenza virus infection. J. Med. Virol..

[20] Huang W.J., Cheng Y.H., Tan M.J., Liu J., Li X.-Y., Zeng X.-X., Tang J., Wei H.-J., Chen T., Yang L. (2022). Epidemiological and virological surveillance of influenza viruses in China during 2020–2021. Infect. Dis. Poverty.

[21] Feng L., Zhang T., Wang Q., Xie Y., Peng Z., Zheng J., Qin Y., Zhang M., Lai S., Wang D. (2021). Impact of COVID-19 outbreaks and interventions on influenza in China and the United States. Nat. Commun..

[22] Hale T., Angrist N., Goldszmidt R., Kira B., Petherick A., Phillips T., Webster S., Cameron-Blake E., Hallas L., Majumdar S. (2021). A global panel database of pandemic policies (Oxford COVID-19 Government Response Tracker). Nat. Hum. Behav..

[23] Magklara K., Kyriakopoulos M. (2023). The impact of the COVID-19 pandemic on children and young people. Psychiatriki.

[24] Huang Q.S., Wood T., Jelley L., Jennings T., Jefferies S., Daniells K., Nesdale A., Dowell T., Turner N., Campbell-Stokes P. (2021). Impact of the COVID-19 nonpharmaceutical interventions on influenza and other respiratory viral infections in New Zealand. Nat. Commun..

[25] Li T., Chen N., Wang X., Chen N., Jin Z., Fang P., Li X., Liu X., Zhu Z., Yang J. (2023). Epidemiology of influenza A outbreak among children after COVID-19: A single-center retrospective observational study. J. Med. Virol..

[26] Ryu S., Cowling B.J. (2021). Human Influenza Epidemiology. Cold Spring Harb. Perspect. Med..

[27] Dave K., Lee P.C. (2019). Global Geographical and Temporal Patterns of Seasonal Influenza and Associated Climatic Factors. Epidemiol. Rev..

[28] Koel B.F., Burke D.F., Bestebroer T.M., van der Vliet S., Zondag G.C.M., Vervaet G., Skepner E., Lewis N.S., Spronken M.I.J., Russell C.A. (2013). Substitutions near the receptor binding site determine major antigenic change during influenza virus evolution. Science.

