# Supplementary material for: Epidemiology and Genetic Evolutionary Analysis of Influenza Virus Among Children in Hainan Island, China, 2021–2023
Source: Pathogens. 2025 Feb 3;14(2):142. doi: 10.3390/pathogens14020142 (PMC11858400; doi:10.3390/pathogens14020142)
Supplement: Supplementary file 1 [file pathogens-14-00142-s001.zip › pathogens-3416076-supplementary.pdf]

Table S1 Primers and PCR conditions for the HA and NA regions

**Name**	**Sequence (5'-3')**	**Annealing temperature (℃)**
H1N1-HA-F	AGCAAAAGCAGGGGAAAATAAAAGC	55
H1N1-HA-R	CCTACTGCTGTGAACTGTGTATTC	
H1N1-NA-F	AGCAAAAGCAGGAGTTTAAAATG	55
H1N1-NA-R	CCTATCCAAACACCATTGCCGTAT	
H3N2-HA-F	TGCATCACTCCAAATGGAAGCATT	55
H3N2-HA-R	ATATCGTCTCGTATTAGTAGAAACAAGGGTGTTTT	
H3N2-NA-F	CATGCGATCCTGACAAGTGTTATC	55
H3N2-NA-R	ATATGGTCTCGTATTAGTAGAAACAAGGAGTTTTTT	
IBV-HA-F	ATAGGAAATTGCCCAATATGGGT	55
IBV-HA-R	CGTTTCTTTGTAATGATGACAAG	
IBV-NA-F	ACTTATTTGGACACCCCCAGACC	55
IBV-NA-R	AGTAGTAACAAGAGCATTTTTCAGA	
